# The emptied bacteriophage T4 head: a novel nanoparticle vehicle to simultaneously deliver HIV-1 antigens and genes into dendritic cells

**DOI:** 10.1186/1742-4690-9-S2-P324

**Published:** 2012-09-13

**Authors:** V Rao

**Affiliations:** 1 The Catholic University of America, Washington, DC, USA

## Background

Simultaneous delivery of HIV-1 antigens and genes by a single nanoparticle will greatly aid in the development of effective HIV-1 vaccines. We have created a highly stable, empty bacteriophage T4 capsid shell which can be engineered both inside and out. The empty head can be filled with foreign DNA using a powerful DNA packaging machine. Its surface can be arrayed with proteins as fusions of the outer capsid proteins, Hoc (highly antigenic outer capsid protein) and Soc (small outer capsid protein). The 120 x 86 nm head has a capacity to accommodate 170-kb foreign DNA and 1025 molecules of proteins.

## Methods

A “neck” mutant fails to seal the packaged head of phage T4. The packaged viral genome is spontaneously emptied to create empty space inside. The pentameric packaging motor is assembled at the portal vertex. A variety of genes; reporter genes luciferase and GFP, HIV-1 envelope genes gp41, gp145, and gp160, and/or molecular adjuvant gene GMCSF are packaged inside the head. The surface is arrayed with enzymes such as the tetrameric β-galactosidase or trimers of HIV-1 gp145 as fusions of Soc, and dendritic cell targeting ligands such as Dec205 mAb or CD40L as fusions of Hoc.

## Results

The emptied phage T4 head efficiently packaged foreign DNA. Packaging is highly promiscuous requiring no specific sequence. Long concatemers, plasmid DNAs, or short PCR-amplified DNAs are packaged efficiently. Multiple genes and multiple copies of each gene are packaged into the same head. The surface is decorated with HIV-1 gp145 trimers and dendritic cell targeting ligands Dec205 mAb or CD40 ligand. The engineered head delivered the massive “payload” into dendritic cells to near 100% efficiency. The delivered genes were abundantly expressed as analyzed by immunoelectron microscopy.

## Conclusion

A novel bacteriophage T4 nanoparticle efficiently delivers DNA-inside, protein-outside, prime-boost HIV-1 vaccines to dendritic cells.

